# Biosynthesis and Properties of a P(3HB-*co*-3HV-*co*-4HV) Produced by *Cupriavidus necator* B-10646

**DOI:** 10.3390/polym14194226

**Published:** 2022-10-09

**Authors:** Natalia O. Zhila, Kristina Yu. Sapozhnikova, Evgeniy G. Kiselev, Ivan V. Nemtsev, Anna V. Lukyanenko, Ekaterina I. Shishatskaya, Tatiana G. Volova

**Affiliations:** 1 Institute of Biophysics SB RAS, Federal Research Center “Krasnoyarsk Science Center SB RAS”, 50/50 Akademgorodok, Krasnoyarsk 660036, Russia; 2 Basic Department of Biotechnology, School of Fundamental Biology and Biotechnology, Siberian Federal University, 79 Svobodnyi Av., Krasnoyarsk 660041, Russia; 3 L.V. Kirensky Institute of Physics SB RAS, Federal Research Center “Krasnoyarsk Science Center SB RAS”, 50/38 Akademgorodok, Krasnoyarsk 660036, Russia; 4 Federal Research Center “Krasnoyarsk Science Center of the Siberian Branch of the Russian Academy of Sciences” 50 Akademgorodok, Krasnoyarsk 660036, Russia

**Keywords:** *Cupriavidus necator* B-10646, γ-valerolactone, biomass concentration, content and composition of copolymers, P(3HB-*co*-3HV-*co*-4HV), physicochemical properties, films, surface properties

## Abstract

Synthesis of P(3HB-*co*-3HV-*co*-4HV) copolymers by the wild-type strain *Cupriavidus necator* B-10646 on fructose or sodium butyrate as the main C-substrate with the addition of γ-valerolactone as a precursor of 3HV and 4HV monomers was studied. Bacterial cells were cultivated in the modes that enabled production of a series of copolymers with molar fractions of 3HV (from 7.3 to 23.4 mol.%) and 4HV (from 1.9 to 4.7 mol.%) with bacterial biomass concentration (8.2 ± 0.2 g/L) and PHA content (80 ± 2%). Using HPLC, DTA, DSC, X-Ray, SEM, and AFM, the physicochemical properties of copolymers and films prepared from them have been investigated as dependent on proportions of monomers. Copolymers are characterized by a reduced degree of crystallinity (C_x_ 38–49%) molecular weight characteristics M_n_ (45–87 kDa), and M_w_ (201–248 kDa) compared with P(3HB). The properties of the films surface of various composition including the porosity and surface roughness were studied. Most of the samples showed a decrease in the average pore area and an increase in their number with a total increase in 3HV and 4HV monomers. The results allow scaling up the productive synthesis of P(3HB-*co*-3HV-*co*-4HV) copolymers using *Cupriavidus necator* B-10646.

## 1. Introduction

Polyhydroxyalkanoates (PHA) are actively studied degradable biopolymers intended to solve the problems associated with the global accumulation of plastic waste in the biosphere and with the elimination of deficit in functional materials for special purposes [1,2,3,4]. PHA is a family of polymers of various compositions whose physicochemical properties (molecular weight, crystallinity, degradation rates in biological media) vary significantly depending on the chemical structure [5,6,7,8,9,10,11,12]. Identification of microorganisms and conditions that make it possible to obtain polymers of various chemical compositions is a necessary condition for the development of methods for the synthesis of new polymers with desired properties.

In the fundamental work of Professor Alexander Steinbüchel [13], an idea of the diversity of PHA, the mechanism of their formation, and the nature of microorganism-producers was given. Based on the length of the carbon chain of the hydroxy acids that form polymers, polyhydroxyalkanoates are divided into three main groups: short-chain (short-chain-length, SCL), consisting of acids with a carbon chain length from three to five carbon atoms; medium-chain-length (MCL), which contain from 6 to 14 carbon atoms and long-chain (long-chain-length, LCL) containing acids C_17_ and C_18_. LCL-PHAs are the least common and the most poorly studied PHAs [14]. One of the difficulties in producing such PHAs is related to the carbon source: alkanes and alkanoic acids are water insoluble, and other substrates for the synthesis of LCL-PHAs are toxic for bacteria at relatively low concentrations [15]. Synthesis of small amounts of LCL-PHAs was reported for *Pseudomonas* species [15,16,17], and such strains as *C. necator* IPT027, *Burkholderia cepacia* IPT400 [18], and *Bacillus thuringiensis* EGU45 [19].

The list of studied PHAs is growing, so the ideas about the diversity of PHAs are constantly expanding. It was proposed to subdivide these valuable polymers into two categories depending on the frequency of occurrence: “usual” and “unusual” polyhydroxyalkanoates [20]. Polymers synthesized intracellularly by microorganisms as reserve macromolecules are proposed to be referred to as “usual“ PHAs. This group includes polymers consisting of monomers (e.g., (*R*)-3-hydroxypropionate, (*R*)-3-hydroxybutyrate, (*R*)-3-hydroxyvalerate, (*R*)-3-hydroxyhexanoate, (*R*)-3-hydroxyoctanoate, (*R*)-3-hydroxydecanoate, and (*R*)-3-hydroxydodecanoate, or combinations thereof), which are formed from various carbon sources (sugars, alkanes, aliphatic fatty acids, triacylglycerols, etc.) through conventional routes (usually synthesis and β-oxidation of fatty acids), including the synthesis of (*R*)-3-hydroxyacyl-CoAs. In the final step, these thioethers polymerize to poly[(*R*)-3-hydroxyalkanoates].

The term “unusual” PHAs (UnPHAs) encompasses many different PHAs, including semi-synthetic and synthetic PHAs, homopolymer and copolymer PHAs, and polymers obtained by physical modifications of natural polymers (polymer blends). Among them are PHAs of microbial origin, which are synthesized either from natural monomers bearing various functional groups, or from their chemical derivatives, as well as polymers obtained either by chemical synthesis or by chemical modification of natural polymers.

PHA synthesis involves key enzymes: β-ketothiolase (β-KT), acetoacetyl-CoA reductase (AA-CoA reductase), and PHA synthase. Theyare under the control of the following genes: *phaA*, *phaB*, and *phaC* respectively. In *Cupriavidus necator *(paнee *Hydrogenomonas eutropha*, *Alcaligenes eutrophus*, *Ralstonia eutropha*, and *Wautersia eutropha* [21,22]), these genes are linked in the *phaCAB* operon under one promoter. The conditions that provide a change in the direction of cell anabolism from a protein program towards the synthesis and accumulation of P(3HB) are determined by the redox state of the cytoplasm, the intracellular concentration of pyruvate and free CoA [23]. Under optimal conditions for growing producers, pyruvate, and reducing equivalents in the form of NADH and NADPH are consumed mainly in the tricarboxylic acid cycle for the formation of amino acids, as well as energy conversion in the cell; while maintaining a high level of free CoA, which is an obstacle to the synthesis of the polymer. With unbalanced growth, for example, in the absence of nitrogen in the medium or oxygen deficiency, pyruvate is not included in the tricarboxylic acid cycle but undergoes decarboxylation with the formation of acetyl-CoA, while the level of free CoA is low. This is a favorable condition for the activation of enzymes of the P(3HB) cycle. To date, the biosynthesis of PHA has been fairly well studied. In a review publication by Professor Chen [5], eight possible biochemical pathways for the synthesis of PHA on various C-substrates are described.

The existence of a wide range of PHA-producing microorganisms capable of using various C-substrates for growth is the basis for obtaining various types of PHA with different valuable properties for various applications [24,25,26,27,28,29].

To the unusual and scantily studied types of PHA belong copolymers, containing in addition to 3-hydroxybutyrate (3HB) and 3-hydroxyvalerate (3HV), also 4-hydroxyvalerate (4HV) monomers. Unlike the P(3HB) homopolymer which is characterized by brittleness and thermal instability, the presence of 3HV and 4HV units in copolymers improves their mechanical properties, facilitating processing into products and thereby expanding the potential of applications [30,31,32]. Moreover, due to the presence of amorphous domains in such copolyesters, they are more prone to biodegradation in vivo than P(3HB) [32]. It has been shown that 3HV- and 4HV-containing copolymers exhibit lower melting point and crystallinity degree, as well as greater flexibility [33,34,35,36]. Therefore, increasing the proportion of inclusions of 3- and 4-hydroxyvalerate monomers is an important task, the solution of which will expand the scope of PHA.

In contrast to the well-studied P(3HB-*co*-3HV) two-component copolymers, which are synthesized pure cultures of microorganisms, for example *C. necator* or *Burkholderia sacchari*, use a variety of C-substrates and precursor compounds [37,38] or mixed microbial cultures (MMC) [39,40]. Information on the conditions for the synthesis of P(3HB-*co*-3HV-*co*-4HV) three-component copolymers is very limited (Table 1).

As follows from the data in Table 1, the available literature contains a small number of works devoted to the biosynthesis of P(3HB-*co*-3HV-*co*-4HV) copolymers using γ-valerolactone as a 4HV precursor [41,42,43]. The relationship between the monomer ratio and the 4HV monomer content of these PHAs has been studied even less. The available fragmentary and not free from contradictions data show that the features of these representatives of the PHA family are at a lower glass transition temperature and melting point, as well as a lower degree of crystallinity, which tends to decrease even with small inclusions of 4HV monomers [43,44,46].

Of no less importance for characterization of PHAs is the surface structure of the films produced from them. At the same time, the literature data on the surface structure of the P(3HB-*co*-3HV-*co*-4HV) films are lacking. The significance of the present study is determined by the interdisciplinary approach used in tissue engineering to construct cell culture scaffolds. Biological properties of biomedical materials and devices can be controlled by varying the physicochemical properties of their surfaces (chemical composition, porosity, roughness, morphology, etc.). All of these factors will eventually determine the properties of polymer products, leading to an increase in their biodegradation rates, improvement of their ductility and mechanical strength, and enhancement of their surface hydrophilicity and porosity, which favors cell attachment, improves gas-dynamic properties of the scaffolds, and makes them more permeable for substrates. The literature data on the effects of PHA composition on the properties of the PHA-based products are limited. The present study is the first to report data on the surface structure of P(3HB-*co*-3HV-*co*-4HV) films. Previous studies, however, showed that the properties of P(3HB) nonwoven mats were mainly determined by the properties of the polymer, which, in turn, were determined by the carbon substrate type (e.g., different sugars) [49,50]. Physical/mechanical properties of solution cast PHA films and the surface structure of those films varied considerably depending on the degree of crystallinity of the polymer, which, in turn, was determined by PHA composition and the carbon source used (sugars, glycerol, fatty acids, plant oils) [51,52,53].

The purpose of this work is to study the conditions for the synthesis of P(3HB-*co*-3HV-*co*-4HV) copolymers with different ratios of monomers in the culture of the wild-type strain *Cupriavidus necator* B-10646 and the physicochemical properties depending on the chemical composition.

## 2. Materials and Methods

### 2.1. Microorganisms

The studies were carried out in the culture of a wild-type strain *Cupriavidus necator* B-10646, registered in the Russian National Collection of Industrial Microorganisms (NCIM) [54]. The strain synthesizes PHA copolymers composed of short- and medium-chain-length monomers [52].

### 2.2. Culture Medium and Cultivation Conditions

The collection culture was grown in the mineral Schlegel medium [55]—a strong phosphate-buffered solution. The main carbon source was fructose (Panreac, Barcelona, Spain) or butyric acid (Panreac, Barcelona, Spain) which was added in the medium as sodium butyrate. They were sterilized by membrane filtration (Opticap XL300 Millipore Express SHC filters, Merck, Darmstadt, Germany). To synthesize PHA copolymers, the cell culture was supplemented with precursors of γ-valerolactone (Sigma-Aldrich, Saint Louis, MO, USA). In a number of experiments, the culture medium was also supplemented with sodium acrylate (Sigma-Aldrich, Saint Louis, MO, USA) (simultaneously with γ-valerolactone supplementation). Butyric acid and acrylic acid were neutralized with a 33% NaOH solution (pH 7.0 ± 0.2).

Cells were grown in the batch culture in the mode previously developed for PHA synthesis [56]. Inoculum was produced using an Innova^®^ 44 constant temperature incubator shaker (New Brunswick Scientific, Edison, NJ, USA). Inoculum was prepared by resuspending the stock culture maintained on agar medium. The stock culture was grown in 0.5-L to 1-L glass flasks half-filled with mineral solution, with the initial concentration of fructose (10–15 g/L) or sodium butyrate (2 g/L). Concentration of fructose was determined using the resorcinol method [57].

Concentration of γ-valerolactone was measured after it was extracted from the culture medium, using a 7890A chromatograph-mass spectrometer (Agilent Technologies, Santa Clara, CA, USA) equipped with a 5975C mass detector (Agilent Technologies, Santa Clara, CA, USA). The yields of the culture (Y_x_, g biomass/g substrate) and polymer (Y_p_, g PHA/g substrate) were calculated.

### 2.3. PHA Recovery from Cell Biomass

PHA was extracted from the cell biomass, which was previously separated from the culture fluid by centrifugation in an AvantyJ-HC centrifuge (BeckmanCoulter, Indianapolis, IN, USA). Then, the biomass was dried in an LP10R freeze dryer (ilShinBioBase, Dongducheon-si, Korea) to a residual moisture content of 5%. All solvents were chemically pure and purchased from “Ekos-1” (Staraya Kupavna, Russia). The extraction was carried out in two stages: at the first stage, the biomass was degreased with ethanol; at the second stage the polymer was extracted with dichloromethane. The dichloromethane extracts were evaporated using an R/210V rotary evaporator (Büchi, Flawil, Switzerland). Then, polymer was precipitated with hexane, 1:2. Polymer was re-dissolved in chloroform several times and precipitated using isopropanol or hexane to purify it. The resulting polymer was dried at 40 °C [58].

### 2.4. PHA Chemical Composition

Intracellular content of the polymer and its composition were determined by chromatography of methyl esters of fatty acids after methanolysis of cell biomass using a 7890A chromatograph-mass spectrometer (Agilent Technologies, Santa Clara, CA, USA) equipped with a 5975C mass detector (Agilent Technologies, Santa Clara, CA, USA). Purity of the polymer and its composition were determined by chromatography of methyl esters of fatty acids after methanolysis of purified polymer samples using a chromatograph-mass spectrometer. Methanolysis of the samples was conducted as follows: 1 mL chloroform, 0.85 mL methanol, and 0.15 mL concentrated sulfuric acid were added to a 4.0–4.5 mg polymer sample and boiled under reflux condensers for 160 min. At the end of methanolysis reaction, 1 mL distilled water was added to the flask. The bottom chloroform layer was used for analysis by chromatography [59,60].

### 2.5. Physicochemical Properties of PHAs

Physicochemical properties of PHAs were examined using size-exclusion chromatography, X-Ray structure analysis, and differential scanning calorimetry; methods and instruments have been described in detail elsewhere [52].

Molecular-weight properties of PHAs were examined with a size-exclusion chromatography (Agilent Technologies 1260 Infinity, Waldbronn, Germany), a DB-35MS column was used. Weight average molecular weight (M_w_), number average molecular weight (M_n_), and polydispersity (Ð) were measured using polystyrene standards (PS) (Agilent Technologies, Santa Clara, CA, USA).

Thermal analysis of PHA specimens was performed using a DSC-1 differential scanning calorimeter (Mettler Toledo, Schwerzenbac, Switzerland) and TGA (Mettler Toledo, Schwerzenbac, Switzerland). Thermograms were analyzed using the “STARe v11.0.” software (Mettler Toledo, Schwerzenbac, Switzerland). Crystallization temperature (T_cryst_), glass transition temperature (T_g_), melting point (T_melt_), and thermal degradation temperature (T_degr_) were determined.

X-Ray structure analysis was performed to determine crystallinity of copolymers employing a D8 ADVANCE X-Ray powder diffractometer equipped with a VANTEC fast linear detector (Bruker AXS, Karlsruhe, Germany). The degree of crystallinity (C_x_) was calculated as a ratio of the total area of crystalline peaks to the total area of the radiogram (the crystalline + amorphous components). Calculations were done by using the Eva program of the diffractometer software.

### 2.6. Production and Investigation of Polymer Films

Films of PHAs were prepared by casting chloroform solutions (2%) heated to 35 °C on degreased glass and subsequent drying for 48 h in a dust-free box (Labconco, Kansas, MO, USA).

The surface microstructure of PHA films was analyzed using scanning electron microscopy (FE-SEM S-5500 high-resolution scanning electron microscope Hitachi, Tokyo, Japan). Prior to microscopy, the samples were sputter coated with platinum (at 25 mA, for 60 s), using an EM ACE200 (Leica, Vienna, Austria). The structural elements of the film surface areas formed (grooves or holes, their dimensions, and area), and porosity of the films was determined from SEM images using a software package for digital image analysis (free open-source software package for scientific analysis, editing, and processing of raster images), Image J v1.53k.

The roughness of film surface was determined using atomic-force microscopy (AFM) in semicontact mode (DPN 5000, NanoInk, Skokie, IL USA). The arithmetic mean surface roughness (S_a_) and the root mean square roughness (S_q_) were determined based on 10 points, as the arithmetic averages of the absolute values of the vertical deviations of the five highest peaks and lowest valleys from the mean line of the surface profile, using conventional equations [61]. AFM data were processed, and statistical analysis of the images was performed using the Gwyddion (2.51) free software. Sites of areas 20 × 20 µm were examined, and 2 × 2 µm sites with local maxima and minima were selected; they were analyzed at a higher resolution, and the roughness of each sample was calculated as the average of three measurements.

### 2.7. Statistics

Statistical analysis of the results was performed by conventional methods, using the standard software package of Microsoft Excel. Each experiment was performed in triplicate. Arithmetic means and standard deviations were found. The statistical significance of results was determined using Student’s *t*-test (significance level: *p* ≤ 0.05).

## 3. Results and Discussion

The study of the synthesis of PHA containing 4-hydroxyvalerate (4HV) monomers was carried out in a culture of the highly productive wild-type strain *Cupriavidus necator* B-10646. This strain is characterized by the ability to synthesize polyhydroxyalkanoates of various chemical compositions on sundry substrates (under autotrophic and heterotrophic conditions) and is tolerant to a number of precursor substrates necessary for the synthesis of multicomponent PHAs [52].

The synthesis of PHA copolymers with high polymer content and its monomeric composition is a complex biotechnological problem that depends on many factors. For the synthesis of PHAs with a different set of monomers, it is necessary to introduce into the medium, in addition to the main carbon substrate, also substrates—precursors of the target monomers, which, as a rule, inhibit the growth of producer strains. Therefore, it is necessary to know the boundaries of the physiological action for each studied strain, not only for the main C-substrate, but also for a specific substrate-precursor, taking into account the effect of its concentration and dosing regimes on the production parameters of the culture (concentration of bacterial biomass, accumulation of the polymer in cells, composition and the ratio of monomers in it).

### 3.1. Synthesis of P(3HB-*co*-3HV-*co*-4HV) Copolymers by Bacteria Cupriavidus Necator B-10646

As a precursor necessary for the synthesis of 4HV monomers, γ-valerolactone was studied, which, along with other substrates (4-hydroxyvaleric or levulinic acids), is used for the synthesis of multicomponent PHA containing 3HV and 4HV monomers [41,42,43].

**Figure 1:**
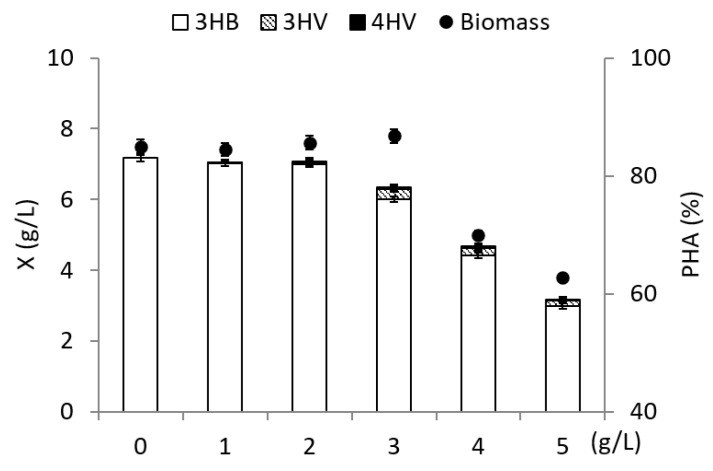
Biomass concentration of *Cupriavidus necator* B-10646, content, and composition of PHA in experiments with fructose as the main C-substrate and addition of γ-valerolactone at various concentrations supplemented into medium on 24 h of cultivation.

The results of the study of the influence of the concentration of γ-valerolactone on the parameters of the *C. necator* B-10646 culture are illustrated in Figure 1. The precursor substrate was introduced into the culture once at various concentrations during the period of active polymer synthesis for 24 h of the bacteria growing process. It was shown that γ-valerolactone added to the culture during the period of active polymer synthesis at a concentration of 1.0 to 3.0 g/L did not inhibit bacterial growth and polymer synthesis. The bacterial biomass concentration for 72 h was, as a rule, 7.6 ± 0.1 g/L, the polymer content in the cells reached 80 ± 2%. Y_x_ was 0.31 g/g, and Y_p_ was 0.25 g/g.

**Figure 2:**
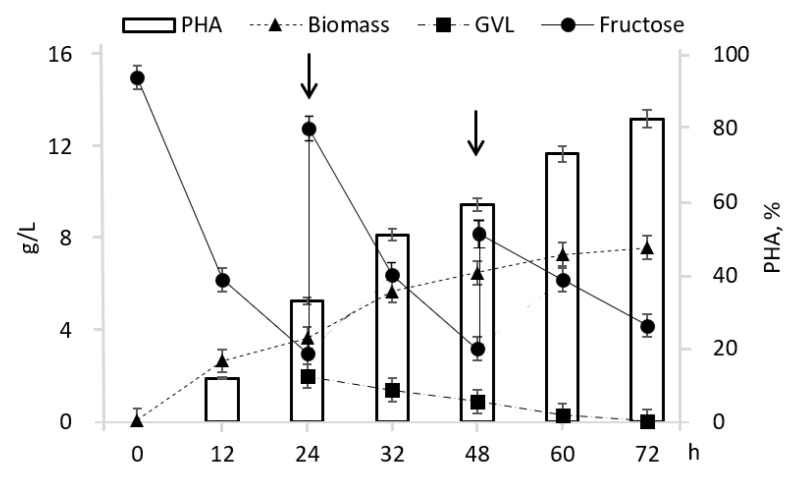
Dynamics of *Cupriavidus necator* B-10646 biomass concentration, polymer content, and fructose and γ-valerolactone concentrations during cultivation. Addition of γ-valerolactone at 24 h of the culture. Arrows point at the addition of fructose during cultivation.

As an example, Figure 2 shows changes in concentrations of fructose and γ-valerolactone (added at a concentration of 2 g/L at 24 h) during cultivation. As fructose was consumed, it was added to reach levels equivalent to its initial concentration at 24 and 48 h. Concentration of γ-valerolactone declined gradually, being reduced to 0.3–0.5 g/L at 24 h and to trace amounts at the end of cultivation. This is comparable to the performance of the control culture grown only on fructose without the addition of γ-valerolactone. A further increase in the concentration of γ-valerolactone above 3.0 g/L inhibited the growth of bacteria, as well as the intracellular accumulation of the polymer, causing a decrease in both parameters.

**Figure 3:**
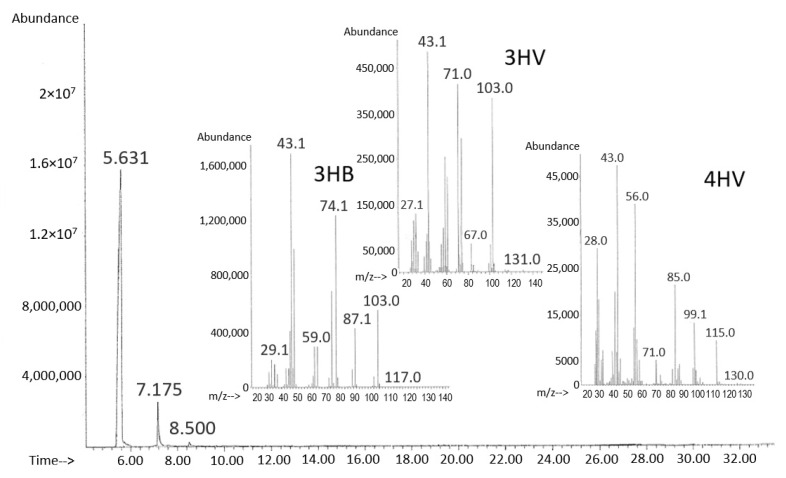
Chromatogram and mass spectra of P(3HB-*co*-3HV-*co*-4HV) sample containing 3HB, 3HV, 4HV monomers. Retention times of monomers, respectively, 3HB, 5.631; 3HV, 7.175; 4HV, 8.500.

To identify the composition of PHA synthesized at different concentrations of γ-valerolactone additions in the culture of *C. necator* B-10646 bacteria, polymer samples isolated from the cell mass and purified to a homogeneous state were subjected to gas chromatography combined with mass spectrometry (Figure 3).

The synthesized samples are three-component copolymers P(3HB-*co*-3HV-*co*-4HV) with a dominant content of 3-hydroxybutyrate (3HB) monomers (about 97.2–99.2 mol.%) and minor inclusions of 3-hydroxyvalerate (3HV) and 4-hydroxyvalerate (4HV) (Figure 1). With the addition of γ-valerolactone at a concentration of 1.0–2.0 g/L, the content of 4HV and 3HV in the copolymer was below 0.5 mol.%. The maximum content of monomers 3HV (2.3 mol.%) and 4HV (0.4 mol.%) was recorded with a single addition of the precursor substrate to the culture at a concentration of 3.0 g/L. At concentrations of γ-valerolactone (4.0 and 5.0 g/L) inhibiting the bacteria growth and synthesis of PHA, the content of these monomers in the copolymer decreased.

An analysis of the literature showed that increasing the content of 4HV monomers in P(3HB-*co*-3HV-*co*-4HV) copolymers is problematic. The authors of well-known works used various strains, various C-substrates and precursors for the synthesis of 4HV monomers (Table 1).

Thus, in the culture of the wild-type strain *A. eutrophus* NCIB 11599, 4-hydroxyvaleric acid was used as a C-substrate at a concentration of 6 g/L. The concentration of the synthesized copolymer P(3HB-*co*-3HV-*co*-4HV) was not high (45%), the content of 4HV monomers was 8.8 mol.%; the content of 3HV and 3HB monomers was at the level of 67.3 and 23.9 mol.%, respectively [41]. In the culture of *Cupriavidus* sp. L7L, when the medium temperature was varied and levulinic acid salt (8.0 g/L) was used, the bacterial biomass concentration did not exceed 3.3–4.0 g/L. The intracellular content of copolymers varied from 53 to 60% in individual experiments; the content of 4HV monomers was at the level of the results obtained in the article presented and ranged from 2.1 to 4.8 mol.%. The content of 3HV monomers varied from 30.5 to 50.1 mol.% and was comparable to the level of 3HB monomers (minimum 45.1, maximum 66.2 mol.%) [46]. In a batch culture of *Cupriavidus necator* ATCC 17699 grown in a 3-L fermenter on glycerol (20 g/L) with the addition of levulinic acid (2.0 g/L), 80% of the polymer was synthesized in 60 h with a total bacterial biomass concentration of 58 g/L; the content of 3HV and 4HV monomers was not much, 18.5 and 2.5 mol %, respectively; 3HB monomers dominated (70.0 mol %). When using levulinic acid as a C-substrate, which was added to the culture of this strain 4 times at a dose of 2.5 g/L for 48 h, a copolymer with a higher content of monomers 4HV (6.7 mol.%) and 3HV (55.3 mol.%) was synthesized [44]. The strain *C. necator* USMAA2-4, when grown on oleic acid with the addition of γ-valerolactone, synthesized a copolymer P(3HB-*co*-3HV-*co*-4HV), in which the content of 3HV monomers varied by an order of magnitude in different experiments (from 9.0 to 91.0 mol.%), while in all variants the content of 4HV monomers did not exceed 1–2 mol.%. The use of γ-valerolactone as the only carbon source did not improve the results; the content of 4HV monomers was 2.0 mol.%, and the content of 3HV monomers was high (94.0 mol.%). At the same time, the production parameters of the culture were low, the biomass concentration and the polymer content were 2.7 g/L and 36%, respectively [43].

In order to increase the content of 4HV and 3HV monomers in the composition of P(3HB-*co*-3HV-*co*-4HV), a series of experiments was carried out with fractional feeding of precursor substrate of *C. necator* B-10646 into the culture. A similar mode of fractional dosing of various precursor substrates was developed earlier and, in combination with a controlled fermentation time of bacteria after additives added to the culture, was successfully implemented earlier [52,56,62,63]. This made it possible to synthesize a wide range of two-, three-, and four-component copolymers with different sets and ratios of 3- and 4-hydroxybutyrate, 3-hydroxyvalerate, and 3-hydroxyhexanoate monomers, the content of which varied from units to 50 mol.% or more, as well as copolymers of complex composition—poly(3-hydroxybutyrate-*co*-3-hydroxyvalerate-*co*-3-hydroxy-4-methylvalerate) [P(3HB-*co*-3HV-*co*-3H4MV)] [64].

**Figure 4:**
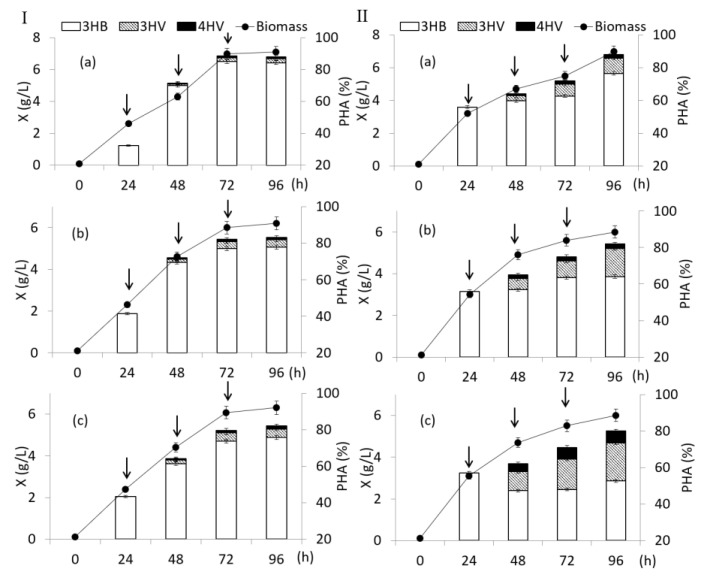
Biomass concentration of bacteria *Cupriavidus necator* B-10646, content and composition of PHA in experiments with fructose (**I**) as the main C-substrate and addition of γ-valerolactone at different concentrations: (**a**)—1.0 + 1.0 + 1.0; (**b**)—1.0 + 1.5 + 1.5; (**c**)—1.5 + 1.5 + 2.0 g/L; (**II**)-under similar conditions with sodium acrylate addition (the time of supplementation is shown by arrows).

The results of the influence of fractional dosing of γ-valerolactone are given in Figure 4 I. A scheme of experiments with γ-valerolactone added in split portions during cell cultivation on fructose is given in Figure S1. A series of experiments was carried out with the cultivation of bacteria *C. necator* B-10646 on fructose with 3 additions of γ-valerolactone to the culture with varying its concentration. In the first experiment, the concentration of the precursor in three additions was 1.0 g/L in each, that is, a total of 3.0 g/L. In the second experiment, the first supplement contained 1.0 g/L γ-valerolactone; the second and third supplements had additives of 1.5 g/L each, in total 4.0 g/L. In the third experiment, the first and second additives contained 1.5 g/L of γ-valerolactone each, the third additive, 2.0 g/L, for a total of 5.0 g/L. The mode of adding additives in all experiments was the same: the first additive for 24 h of the process; the second and third at 48 and 72 h. The intervals between additions were 24 h each. The complexity of the task was that the goal was not only to increase the content of 3HV and 4HV monomers, but also to provide conditions for high overall polymer content and bacterial biomass concentration.

*C. necator* B-10646 culture parameters including biomass concentration and final copolymer content were generally comparable. The highest values were obtained with three additions of γ-valerolactone, 1.0 g/L each. The X value was 7.1 g/L, the copolymer content was 88%. Y_x_ was 0.31 g/g, Y_p_ was 0.27 g/g. In the other two variants, with a total precursor supply of 4.0 and 5.0 g/L, a slight decrease in the biomass concentration (to 6.2–6.3 g/L) was noted while maintaining high PHA content at the level of 82–83%. Y_x_ was 0.32–0.33 g/g, Y_p_ was 0.26–0.27 g/g. The ratio of monomers in the copolymer practically did not change. The 3HB monomers were still dominant (over 90 mol.%); the content of the other two monomers was not high and amounted to 2.9–5.7 mol.% for 3HV and 1.1–2.0 mol.% for 4HV.

A similar tactic of fractional supply of a precursor to a bacterial culture to maximize the synthesis of 4HV monomers in the composition of the P(3HB-*co*-3HV-*co*-4HV) copolymer was used on the example of the wild type strain *A. eutrophus* H16 when grown on γ-valerolactone as the sole carbon source [42]. This substrate was supplied into culture fractionally, with 6 additions at a dose of 1.0 or 2.0 g/L each. The content of 4HV monomers in the synthesized copolymer samples varied from 4.9 to 6.3 mol.%, however, the dominant monomers were 3-hydroxyvalerate (53.9–54.1 mol.%) and 3-hydroxybutyrate (about 40 mol.%). Thus, the fractional supply of precursor substrates did not provide a significant increase in the content of 4HV monomers in the desired copolymers. This suggests that the γ-valerolactone supplied to the culture was not used effectively in the formation of 4HV monomers.

Muzaiyanah and Amirul (2013) when considering the intracellular pathways of utilization of γ-valerolactone, showed that one of the possible pathways for the utilization of this C-substrate can be the conversion of 4-ketovaleryl-CoA to pyruvate and acetyl-CoA in the reactions of the β-oxidation cycle [43]. From this, it should be assumed that blocking the β-oxidation pathway can lead to a more efficient use of γ-valerolactone for the synthesis of 4HV and 3HV monomers and an increase in their content in the composition of the P(3HB-*co*-3HV-*co*-4HV) copolymer. Acrylic acid is known to block fatty acid β-oxidation cycle reactions by inhibiting 3-ketoacyl-CoA thiolase. For example, the use of acrylate in the medium promoted the synthesis of medium-chain copolymers by *Ralstonia eutropha* bacteria on a medium with octanoate additives [65]. It is also known that the addition of sodium acrylate led to an increase in 3HHx monomers to 12–20 mol.% in the *C. necator* B-10646 culture during the synthesis of P(3HB-*co*-3HHx) copolymers with the addition of potassium hexanoate [66]. A similar positive effect of using acrylate to increase the content of medium-chain monomers 3HO and 3HD in copolymers was observed in the *P. putida* KT2440 culture by the authors of [67,68].

Therefore, the next cultivation regime for *C. necator* B-10646 was the simultaneous fractional addition of sodium acrylate and γ-valerolactone to the culture at concentrations that did not inhibit bacterial growth and polymer accumulation (Figure 4II). A scheme of experiments with γ-valerolactone and sodium acrylate added in split portions during cell cultivation on fructose is given in Figure S1. Previously, a high inhibitory activity of sodium acrylate against the studied strain *C. necator* B-10646 was shown [66] and it was found that the non-inhibitory values of this substrate do not exceed 0.35 g/L.

In this regard, for this mode of cultivation, a single addition of sodium acrylate did not exceed 0.1 g/L. Additives of sodium acrylate were carried out simultaneously with the introduction of γ-valerolactone according to the strategies described above (Figure 4II). The polymer content in all experiments was close and was at least 80%. At the end of cultivation, the biomass concentration with the addition of 3.0, 4.0, and 5.0 g/L of γ-valerolactone was 7.0, 6.0, and 6.0 g/L, respectively. Y_x_ was 0.31–0.32 g/g, Y_p_ was 0.25–0.26 g/g. This made it possible to increase the content of 4HV and 3HV monomers, respectively: to 2.7 and 10.4 mol.% with a total supply of 3.0 g/L of γ-valerolactone to the culture; up to 3.0 and 18.8 mol.% when feeding the precursor at a concentration of 4.0 g/L. When 5.0 g/L of γ-valerolactone was added to the culture, the content of 4HV and 3HV monomers increased to 8.0 and 26.2 mol.%.

Thus, the addition of sodium acrylate, which is an inhibitor of β-oxidation of fatty acids, apparently made it possible to redirect γ-valerolactone directly to the synthesis of 3HV and 4HV monomers; this led to an increase in their content in the copolymer.

**Figure 5:**
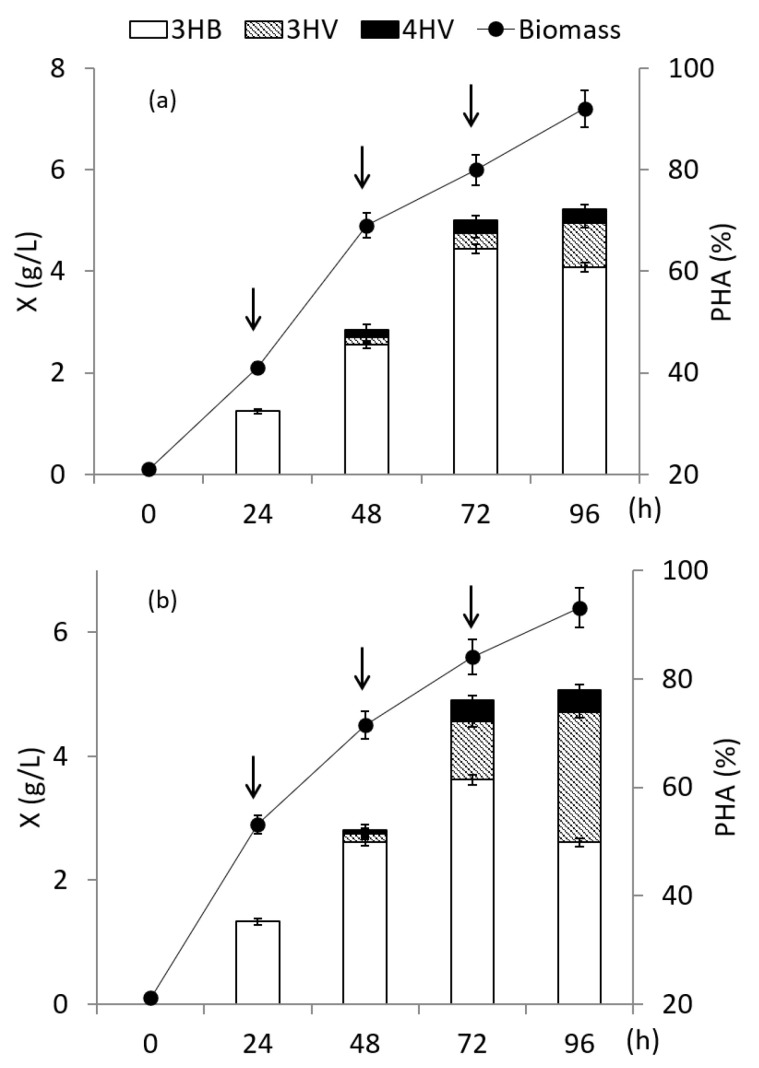
Biomass concentration of bacteria *Cupriavidus necator* B-10646, content and composition of PHA when sodium butyrate is used as the main C-substrate with addition of γ-valerolactone at different concentrations: (**a**)—1.0 + 1.0 + 1.0; (**b**)—1.5 + 1.5 + 2.0 g/L (the time of supplementation is shown by arrows).

The main carbon source can also affect the PHA composition [6,7,12]. Therefore, the synthesis of P(3HB-*co*-3HV-*co*-4HV) in *C. necator* B-10646 culture was studied by replacing fructose with sodium butyrate as the main carbon source. The second substrate was the addition of γ-valerolactone, which was fractionally added to the culture similarly to previous experiments (Figure 5). A scheme of experiments with γ-valerolactone added in split portions during cell cultivation on sodium butyrate is given in Figure S1.

The current concentration of sodium butyrate in the culture was maintained at the level of 1.5–2.0 g/L, introducing additions of this substrate as it was exhausted. The total amount of additions of γ-valerolactone in the first experiment was 3.0 g/L (Figure 5a), in the second experiment it was increased to 5.0 g/L (Figure 5b). The concentration of bacterial biomass and the final content of the copolymer by the end of cultivation in the first experiment were 6.4 g/L and 72%; in the second experiment slightly increased to 7.2 g/L and 78%, respectively. Y_x_ was 0.69 g/g and 0.72 g/g, Y_p_ was 0.50 g/g and 0.56 g/g respectively. The replacement of fructose with sodium butyrate resulted in a significant increase in the content of 4HV and 3HV monomers in the copolymer. So, when applying γ-valerolactone at a total concentration of 3.0 g/L, the content of 4HV monomers increased to 3.6 mol.%, and the content of 3HV monomer (up to almost 11 mol.%) compared with the results obtained using fructose in similar modes growing bacteria. An even higher content of these monomers was achieved by increasing the supply of γ-valerolactone to the cultures at a concentration of 5.0 g/L. The content of 3HV and 4HV monomers was 30.7 and 5.4 mol.%, respectively. The addition of sodium acrylate to a bacterial culture grown on sodium butyrate had no positive effect on the synthesis of 3HV and 4HV copolymers and their content in the copolymer, in contrast to the effect on fructose.

Thus, the regimes that were determined and implemented ensured an increase in the content of 4HV (up to 5.4 mol.%) and 3HV (up to 30.7 mol.%) monomers in the composition of the copolymer synthesized by the wild type strain *C. necator* B-10646, while maintaining the parameters for the concentration of bacterial biomass and the total content of PHA. Higher content of monomers 4HV (up to 24–47 mol.%) and 3HV (up to 50–76 mol.%) in P(3HB-*co*-3HV-*co*-4HV) was obtained only in cultures of recombinant producers of *P. putida* GPp104 with genes *phaC* and *phaE* synthases from *Thiocapsapfennigii* [69] and *Pseudomonas putida* EM42 with PhaEC synthase genes from *Thiocapsapfennigii* or genes for acetyl-CoA acetyltransferase *phaA* and acetyl-CoA reductase *phaB* from *Cupriavidus necator* [70].

### 3.2. Physicochemical Properties of P(3HB-*co*-3HV-*co*-4HV) Copolymers Depending on the Ratio of Monomers

Variation of cultivation regimes of *C. necator* B-10646 bacteria, including the use of different carbon sources (fructose or sodium butyrate) and different dosing regimens of γ-valerolactone, made it possible to synthesize a series of samples of P(3HB-*co*-3HV-*co*-4HV) copolymers with different ratios monomers 3HB, 3HV, and 4HV and investigate the dependence of the basic physicochemical properties of copolymers (molecular weight, temperature parameters, and degree of crystallinity) on the ratio of monomers in them (Table 2).

An important characteristic of polymers is their molecular weight, which depends on the strain characteristics of producers, cultivation conditions, carbon substrate mode, and other factors, influencing the processing of polymers into products and their physical and mechanical properties. All samples of P(3HB-*co*-3HV-*co*-4HV) had lower values of number average (M_n_) and weight average (M_w_) molecular weight and higher values of polydispersity (Ð) compared to P(3HB). At the same time, the M_n_ and M_w_ values for samples with different contents of 3HV and 4HV monomers were in the ranges of 45–87 and 201–248 kDa, respectively, that is, they differed slightly from each other. The values of Ð were minimal (2.8) for the sample with the lowest content of 4HV monomers (1.9 mol.%); in other samples, Ð was higher, at the level of 3.4–4.9.

Comparison of the obtained results with published ones showed the ambiguity of the influence of the ratio of monomers in the studied copolymers on the molecular weight. Higher M_w_ values (510–570 kDa) were found for several P(3HB-*co*-3HV-*co*-4HV) samples synthesized by the wild type strain *C. necator* USMAA2-4 on oleic acid with additions of γ-valerolactone and containing the same and low inclusions (1–2 mol.%) of 4HV monomers with different amounts of 3HB monomers (27 and 91 mol.%) [43]. However, for other samples from the same series with a similar low content of 4HV monomers at a content of 3HV 55 mol.%, M_w_ was significantly lower, at the level of 110–280 kDa. Higher and similar values of M_w_ (810 and 1060 kDa) are given in [44] when comparing P(3HB-*co*-3HV-*co*-4HV) samples synthesized by the wild type strain *Cupriavidus necator* ATCC 17699 on glycerol with additives of levulinic acid or only with levulinic acid. In the synthesized samples, the content of 3HV monomers was 2.5 and 6.7 mol.%, and the difference in 4HV was more significant (18.5 and 55.3 mol.%), i.e., an increase in 3HV + 4HV monomers did not reduce the molecular weight of the copolymers. Comparison of P(3HB-*co*-3HV-*co*-4HV) samples synthesized by *Cupriavidus sp.* L7L on salts of levulinic acid as the only C-source, in which the content of monomers varied, showed the highest values of M_w_ (3624 and 4032 kDa) with the highest content of monomers 3HV (40.4 and 50.1 mol.%) and 4HV (2.8 and 4.8 mol.%) [46]. The authors of this work observed a decrease in M_w_ with a decrease in the content of 3HV monomers to 33.7 and 30.5 mol.% and 4HV monomers to 2.1 and 3.3. mol.%, respectively. Thus, the results obtained in this work and published data do not allow us to unambiguously interpret the effect of the content of 3HV and 4HV monomers on the molecular weight of P(3HB-*co*-3HV-*co*-4HV).

The temperature characteristics of PHA are significant parameters, since they determine the thermomechanical properties and the possibility of obtaining products from melts. In PHA, the main type of phase equilibrium is the condensed state-crystalline, glassy, viscous, and liquid. As is known, homopolymer P(3HB) is characterized by: softening temperature-about 110 °C, crystallization range (T_cryst_)-85–110 °C; melting point (T_melt_)and thermal degradation temperature (T_degr_) have a gap of at least 100 °C and are in the range of 160–180 and 270–280 °C respectively. As a rule, PHA copolymer samples have lower T_melt_ and T_degr_ [6,7,8,9,10,11,12].

**Figure 6:**
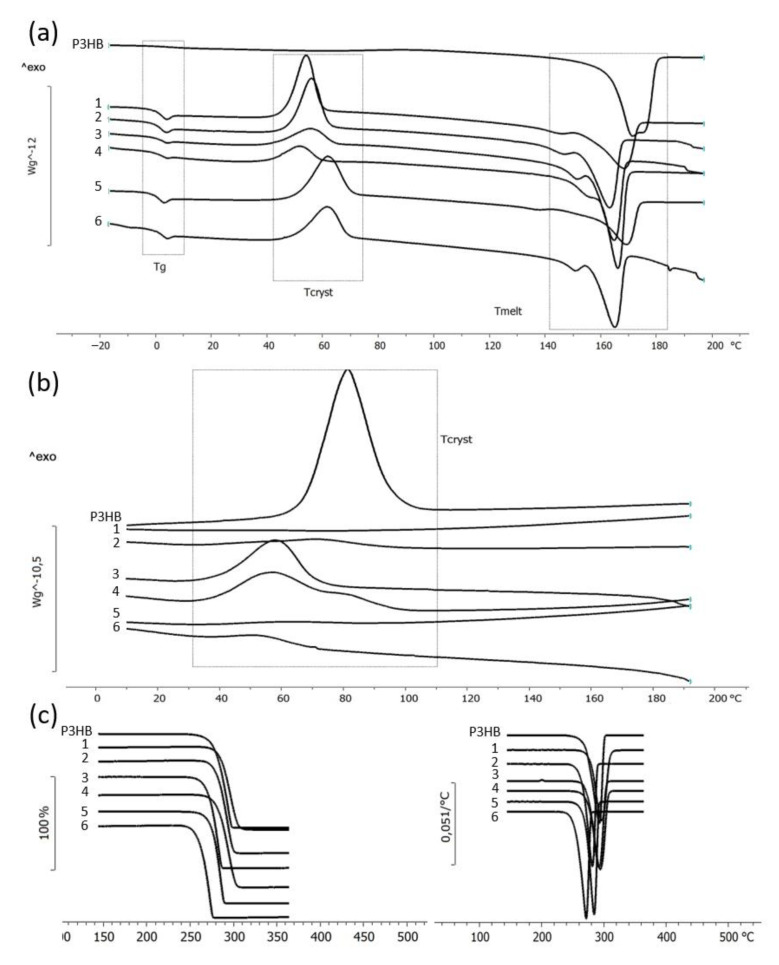
Temperature characteristics of P(3HB-*co*-3HV-*co*-4HV) samples with different sets of monomers: (**a**)-DTA (or DSC) curves with glass transition temperature (T_g_), crystallization temperature (T_cryst_) and melting point (T_melt_) regions; (**b**)-crystallization temperature; (**c**)-thermal stability (TGA).

The results of studying the temperature characteristics of the synthesized P(3HB-*co*-3HV-*co*-4HV) samples are illustrated in Table 2 and Figure 6. All samples of copolymers had reduced T_melt_, T_cryst_, and glass transition temperatures (T_g_) compared to P(3HB) by 10–17 °C. A feature of all samples was the presence of double peaks in the melting region (Figure 6a). For the P(3HB) sample and the copolymer sample with the content of 3HV and 4HV monomers, respectively, 16.3 and 2.5 mol.%, the second melting peak had a small shoulder near the main peak (Figure 6a, sample 4). The lowest T_melt_ value (peaks at 160 and 142 °C) was recorded for the sample with almost the highest content of 3HV monomers (20.8 mol.%) (Figure 6a, sample 5). In contrast, the sample with a similarly high content of 3HV monomers (23.4 mol.%) and the highest content of 4HV monomers (4.7 mol.%) had a slightly higher melting point (melting peaks were recorded at 150.4 and 164.8 °C). For other P(3HB-*co*-3HV-*co*-4HV) samples with a lower content of 3HV monomers and a similar content of 4HV monomers, slightly higher T_melt_ are also characteristic-the first peak was recorded in the region of 145–151 °C; the second peak is at 162.9–168.0 °C (Table 2).

The content of 3HV and 4HV monomers in the copolymers had the most significant effect on the glass transition temperature (Figure 6a). Thus, with an increase in the content of these monomers, the glass transition temperature decreased from 5.3 °C (T_g_ for the P(3HB) homopolymer) to –10.6 °C for the copolymer sample with the highest content of 3HV and 4HV (23.4 and 4.7 mol.%), respectively. This sample is characterized by the presence of two glass transition temperatures (Figure 6a, sample 6), which may indicate the inhomogeneity of the composition of this sample.

All copolymer samples also had a significant reduction in crystallization temperature compared to P(3HB) (Figure 6b). The lowest crystallization temperature was recorded for samples 3 and 4 with a total content of 3HV + 4HV monomers of 16.3 and 18.8 mol.%. For samples with 3HV + 4HV content of 10.1 and 21.1 mol.% (samples 1 and 5, respectively), the effect of prolonged crystallization was recorded (Figure 6b). These samples did not crystallize when cooled under the experimental conditions; their crystallization took place during reheating. The remaining samples of copolymers are characterized by the presence of two crystallization peaks. The first peak was observed when the samples were cooled; the second-during reheating. This may indicate the manifestation of the processes of extended crystallization, as well as the processes of recrystallization and improvement of the processes of crystal formation.

The thermal stability of the P(3HB-*co*-3HV-*co*-4HV) samples was evaluated by TGA (Figure 6c). All samples are characterized by a large range of thermal stability, up to 260 °C. In this case, the degradation temperature of the samples did not directly depend on the composition of the monomers in the copolymer. The highest thermal stability was recorded for the sample with the lowest content of 3HV and 4HV monomers (7.3 and 2.8 mol.%, respectively, 10.1 mol.% in total), in which the thermal degradation temperature was 284.1 °C. This is 5 °C higher than that of the thermostable homopolymer P(3HB) (Figure 6c, sample 1). An increase in thermal stability to 280.4 and 281.2 °C is also characteristic of samples with a total low and medium content of 3HV + 4HV monomers, respectively, 11.7 and 18.9 mol.% (Figure 6c, samples 2 and 4). For the rest of the samples, a slight decrease in thermal stability to 270.6, 275.1 and 263.4 °C was recorded. The largest decrease in the thermal degradation temperature (by 15.6 °C) was recorded for the sample with the highest content of 3HV and 4HV monomers (23.4 and 4.7 mol.%, respectively) (Figure 6c, sample 6). Thus, an increase in the content of monomers other than 3HB monomers in the composition of P(3HB-*co*-3HV-*co*-4HV) reduces the thermal stability of the copolymers.

An analysis of a few publications allows us to conclude that the results on the temperature characteristics of P(3HB-*co*-3HV-*co*-4HV) copolymers are very limited and ambiguous. Thus, for samples with a close content of 4HV monomers (1–2 mol.%) and significant differences in the content of 3HB monomers (9–55 mol.%), the melting point was almost the same (145–147 °C). It turned out that with a significant increase in the content of 3HV (from 9 to 55 mol.%), the temperature of thermal degradation increased slightly (from 145.2 to 148.9 °C), and the glass transition temperature fell by half (from −24.4 to −12.8 °C) [43]. The sample with the highest content of 3HV monomers (91.0 mol.%) had the lowest melting point (95.5 °C). Quite different temperature characteristics and the almost complete absence of dependence on the composition of monomers in P(3HB-*co*-3HV-*co*-4HV) were registered in [46]. At different contents of monomers 3HV (from 30.5 to 50.1 mol.%) and 4HV (from 3.3 to 4.8 mol.%), the melting point had reduced and similar values (about 70–80 °C). At the same time, the temperature of thermal degradation was close (236–256 °C). Thus, the gap between T_melt_ and T_degr_ was significant (over 200 °C); the glass transition temperature was in the ranged from −0.62 to −7.87 °C. In another work [44], samples with different contents of 3HV monomers (from 18.5 to 55.3 mol.%) and 4HV monomers (from 2.2 to 6.7 mol.%), the melting point differed significantly. The highest T_melt_ value was in the sample with the lowest content of 3HV and 4HV monomers, while having three peaks in the region of 79, 161 and 175 °C. The glass transition temperature of the sample was fixed at −1.3 °C. An increase in the content of 3HV and 4HV monomers in 3 other samples was accompanied by a decrease in the melting point to 60–70 °C and two closely lying peaks, as well as a decrease in the glass transition temperature. In a series of P(3HB-*co*-3HV-*co*-4HV) samples synthesized by recombinant *P. putida* GPp104 (pHP1014::E156) and *P. putida* GPp104 (pHP1014::B28RV) strains carrying the *phaC* and *phaE* synthase genes from *Thiocapsapfennigii*, with a higher content of monomers 4HV (18–47 mol.%) and 3HB (50–84 mol.%) at a low content of monomers 3HB (1.5–3.7 mol.%), very low T_melt_ values were recorded with two peaks in the region of 49 and 60, 53 and 79 °C, and almost the same glass transition temperature, about 11.5–12.9 °C [69].

The ambiguity of temperature values is also typical for two-component copolymers P(3HB-*co*-3HV) with different content of 3HV monomers. It was shown that for copolymers of this type with different contents of 3HV monomers (18.0 and 34.7 mol.%) T_melt_ practically did not differ amounting to 167 and 160 °C, respectively. Similar results were obtained by Novackova et al. [47]. For copolymers differing in the content of 3HV monomers by almost three times (27 and 70 mol.%), T_melt_ was close but significantly lower, at the level of 99–103 °C [48]. However, in other papers, T_melt_ samples with different content of 3HV monomers (16 and 53 mol.%) differed significantly and amounted to 150 and 102 °C, respectively [45].

The ability of PHA to crystallize is determined by the internal properties of its chains and is characterized by the crystallization temperature (T_cryst_). The ratio of ordered (crystalline) and disordered (amorphous) phases or the degree of crystallinity (C_x_) is an important characteristic of polymeric materials. PHA are semi-crystalline materials, since the crystallization process does not capture the entire volume of the material. Studies of the degree of crystallinity of PHA are the least explored area of these polymers.

**Figure 7:**
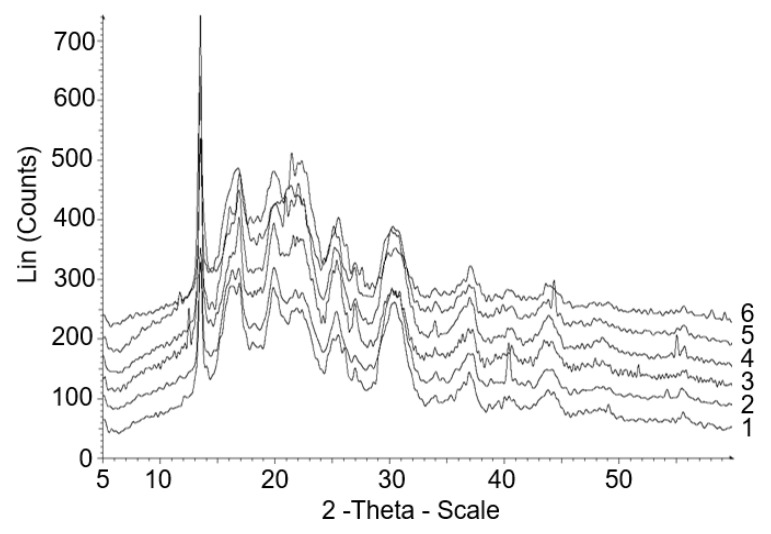
X-ray of P(3HB-*co*-3HV-*co*-4HV) samples with different set of monomers (the numbering indicating the composition of the copolymer is similar to the Table 2).

The results of X-ray diffraction analysis of P(3HB-*co*-3HV-*co*-4HV) of various compositions showed that the degree of crystallinity in all 6 samples is significantly lower than that of the highly crystalline homopolymer P(3HB) (Figure 7, Table 2). The registered C_x_ values for a series of studied samples were below 50%, at the level of 38–49%, i.e., the disordered amorphous phase in the P(3HB-*co*-3HV-*co*-4HV) copolymers prevailed over the crystalline one. The highest degree of crystallinity (49%) was recorded for the sample with the highest content of 3HB monomers (89.9 mol.%) and, accordingly, with the lowest content of 3HV + 4HV monomers (10.1 mol.%); the lowest (38%) was in the sample with the smalest content of 3HB monomers (71.9 mol.%) and the highest content of 3HV (23.4 mol.%) and 4HV (4.7 mol.%) monomers. It should be noted that, in principle, low inclusions of monomers 3HV (not higher than 23 mol.%) and 4HV (not higher than 4.7 mol.%) in total had a very significant effect on the C_x_ P(3HB-*co*-3HV-*co*-4HV) value, reducing it below 50% relative to homopolymer P(3HB). A similar synergistic effect was described in [63]. The authors observed a significant change in the physicochemical properties of three- and four-component PHAs at a relatively low content of 3HV, 4HV, and 3HHx monomers, which in total exceeded the effect of their high concentrations individually on the degree of crystallinity and temperature indices of copolymeric PHAs.

The obtained results on the degree of crystallinity P(3HB-*co*-3HV-*co*-4HV) are consistent with the data of [44]. The authors also registered C_x_ values below 50%, at the level of 34 and 42% for samples with different contents of 3HV (18.5 and 55.3 mol.%) and 4HV (2.2 and 6.7 mol.%) monomers. The amorphization of P(3HB-*co*-3HV-*co*-4HV) samples was recorded to a greater extent by the authors of [43]. In a series of copolymer samples with a close content of 4HV monomers (1–2 mol.%), with an increase in the content of 3HV monomers by an order of magnitude (from 9.0 to 91.0 mol.%), the degree of crystallinity decreased by only twice (from 20.8 to 9.7%).

Copolymers of 3-hydroxybutyrate with 3-hydroxyvalerate are known to be isodimorphic due to the cocrystallization of these monomers [71]. Both monomeric units (3HB and 3HV) have similar shapes and occupy the same volumes. The conformation of the polymer chains of these monomers is compatible with both types of crystal lattice. Changes in the ratio of monomers in P(3HB-*co*-3HV) lead to changes in the crystal lattice. If the content of 3HV monomers in the copolymer is less than 40 mol.%, 3HB monomers can crystallize in the 3HB lattice; if the content of 3HV exceeds 40 mol.%, 3HB monomers can crystallize in the 3HV lattice, that is, isodimorphism affects the level of crystallinity of the copolymer. Previously, it was shown that a decrease in the C_x_ value for P(3HB-*co*-3HV) copolymers is observed in the range of an increase in the content of 3HV monomers only from a few to 25–30 mol.%. For samples with a higher content of 3HV (50–70 mol.%), the degree of crystallinity does not decrease below 50% [62]. This is consistent with the data of other authors [6,7,9]. Thus, the presence in P(3HB-*co*-3HV-*co*-4HV), in addition to 3-hydroxyvalerate monomers, of even small inclusions of 4-hydroxyvalerate monomers enhances the amorphization of P(3HB-*co*-3HV-*co*-4HV) copolymers.

### 3.3. Characteristics of Solvent Cast Films Produced from P(3HB-*co*-3HV-*co*-4HV) Depending on Monomers Ratio

The properties of the surface of polymer products depend on many factors, primarily on the chemical composition of the material used, as well as the method of obtaining products, their dimensions and geometry, etc. Important parameters of cell matrices (scaffolds) are the development of the surface, including porosity, roughness, and hydrophilicity, which together determine adhesive properties, affecting the attachment, differentiation, and development of eukaryotic cells [72]. The influence of the composition of PHA and the methods of processing these polymers on the biological and functional properties of products made from them for biomedical purposes have been described by many authors [73,74,75,76].

**Figure 8:**
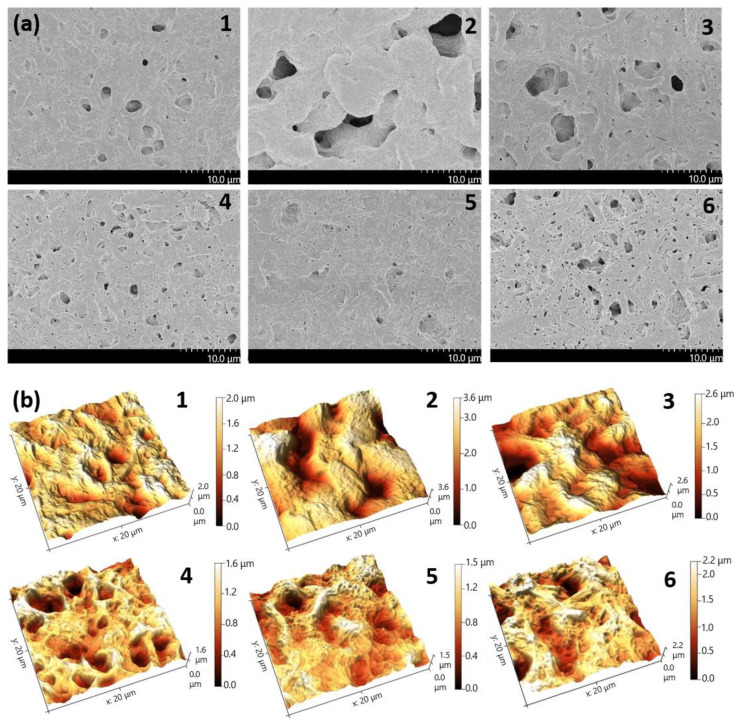
SEM (**a**) and AFM (**b**) images of solvent cast films produced from P(3HB-*co*-3HV-*co*-4HV) of different compositions (the numbering indicating the composition of the copolymer is similar to the Table 2).

The surface properties of solvent cast films produced from P(3HB-*co*-3HV-*co*-4HV) of different compositions were investigated for the first time. The results of SEM and AFM microscopy and characteristics of the porosity and surface roughness of the films are shown in Figure 8 and in Table 3. Films obtained from P(3HB-*co*-3HV-*co*-4HV) samples with different ratios of monomers had differences in the studied parameters, however, without a direct relationship with the composition of monomers in the copolymer. The formation of pores and the formation of a microrelief of the film surface are apparently associated with differences in the crystallization kinetics of the P(3HB-*co*-3HV-*co*-4HV) samples during film formation as the solvent evaporates. On the whole, except for the sample obtained from the copolymer containing 7.3 mol.% 3HV monomers and 2.8 mol.% 4HV, a decrease in the average pore area (from 248.4 to 2.6–5.5 µm^2^) and, on the contrary, an increase in the number of pores (from 2.6 to 88–133.7 pore/1000 µm^2^) with a decrease in the content of the main monomer 3HB from 89.9 to 71.9 mol.% and, accordingly, a total increase in the monomers 3HV and 4HV.

The highest total film porosity (1047 µm^2^/1000 µm^2^), determined by the size and number of pores, was in the sample obtained from the copolymer with an average content of 3HV monomers (14.4 mol.%) and the lowest (1.9 mol.%) of 4HV monomers. This sample is characterized by the presence of the largest pores with an average area of 248.4 µm^2^. At the same time, the number of pores was not high (12.8 pores/1000 µm^2^). The total pores area 488 µm^2^/1000 µm^2^ was recorded for films obtained from a copolymer sample with the highest content of 3HV and 4HV monomers, respectively, 23.4 and 4.7 mol.%, which has the lowest degree of crystallinity (38%). This sample is characterized by the presence of a relatively large number of small pores (88.0 pores/1000 µm^2^) with the average pore area was 5.5 µm^2^. For other samples, in which the total content of 3HV + 4HV monomers varied from 10.1 to 23.1 mol.%, the porosity indices did not differ so much. The total pore area ranged from 300–350 to 500–600 µm^2^/1000 µm^2^. On the whole, the porosity of films made of all types of P(3HB-*co*-3HV-*co*-4HV) is comparable to that of films made of P(3HB-*co*-3HV) copolymers and exceeds by orders of magnitude the porosity of dense and almost pore-free films made of P(3HB) homopolymer (Table 3).

A significant parameter of the properties of cell carriers is roughness, which characterizes the physicochemical reactivity of the surface. Surface roughness at the nanometer level affects protein adhesion, cell attachment, cell growth, and the synthesis of specific proteins. The results of the study of the roughness indices of films obtained from P(3HB-*co*-3HV-*co*-4HV) of different compositions are illustrated in Figure 8 and Table 3. The values of the arithmetic mean roughness (S_a_) were recorded which is similar to the value of the standard deviation (S_q_), the difference is that it is calculated as the sum of the modules of the difference between the data value and the average, instead of the squares of the difference. The integral indicator was the maximum height (S_z_) which includes the full range of values, that is the total difference between the minimum and maximum of the profile irregularities (between the maximum depth of the depression (S_v_) and S_p_ (maximum peak height).

The surface roughness indices of the films obtained from copolymer samples with different ratios of 3HB, 3HV, and 4HV monomers differed, but were less pronounced compared to the porosity indices. In this case, the fluctuations of all roughness indices (S_a_, S_q_, and S_z_) did not directly depend on the content of monomers in various P(3HB-*co*-3HV-*co*-4HV) samples. The highest values of S_a_, S_q_, and S_z_, respectively, 544, 699, and 3994 nm, were noted for copolymers with 3HV content of 9.4 and 4HV of 2.3 mol.%, which are characterized by the largest pore area. For other samples, these parameters were lower, being in the ranges of 202–388, 260–491, and 1738–2878 nm, respectively. This is close to the film roughness indices obtained from P(3HB-*co*-3HV) copolymers and exceeds the data for films obtained from the P(3HB) homopolymer.

Earlier, in a comparative study of the surface properties of polymer films obtained from P(3HB) homopolymer and copolymer PHA with different sets and ratios of 3HB, 4HB, 3HV, and 3HHx monomers, it was shown that all copolymer films have higher roughness, more developed and porous surface, as well as higher elasticity. Samples, obtained from all types of copolymers, had a more pronounced surface relief and increase roughness values (up to 200–300 nm). These samples had a pores, sizes and amount of which were dependent on the composition of PHA, pores quantity increased with increasing of the content of 4HB monomers in sample. P(3HB), against the decrease in mechanical strength of samples, measured by elongationat break and Young’s modulus, which values were lower, than that of P(3HB), by, at minimum of 1.4 times, and, at maximum of 1.8 times (up to 1000–1400 MPa) [52]. It has also been shown that the surface properties of PHA polymer films are affected by the type of carbon source, for example, P(3HB) synthesized using C-substrate of glycerol or sugars (glucose), influenced the properties of polymer films. The P(3HB) specimens synthesized from glycerol had reduced M_w_ (300–400 kDa) and degree of crystallinity (50–55%) compared to the specimens synthesized from glucose (860 kDa and 76%, respectively). The low-crystallinity P(3HB) specimens, regardless of the degree of purity of glycerol, produced a beneficial effect on the properties of polymer films, which had a better developed folded surface and increased hydrophilicity. The values of the highest roughness (R_a_) of the films synthesized from glycerol were 1.8 to 4.0 times lower compared to the films synthesized from glucose (71.75 nm) [51]. When comparing the composition and properties of films from copolymer PHA synthesized on individual sugars (glucose, fructose) or complex sugar-containing media (hydrolysates of plant raw materials), the porosity of films obtained from copolymer PHA synthesized on Jerusalem artichoke hydrolysates depended on the content of monomers 3HV and 4HB, in this case, the average pore size was lower than that of films made from the P(3HB) homopolymer. As for the integral indicator, the total pore area, a different picture is revealed here. The maximum porosity (1047 µm^2^/1000 µm^2^) was noted for copolymers with a minimum inclusion of 4HB, and the minimum one (122 µm^2^/1000 µm^2^) was noted for those with a maximum content of 4HV [77].

## 4. Conclusions

The synthesis of poorly studied P(3HB-*co*-3HV-*co*-4HV) copolymers by the wild- type strain *Cupriavidus necator* B-10646 on fructose or butyric acid as the main C-substrate with the addition of γ-valerolactone as a precursor of the 3HV and 4HV monomers were studied. By varying the bacterial cultivation conditions, including the type of the main C-substrate andthe amount of the precursor substrate added to the bacterial culture in the mode of reserve polymer synthesis, we studied the production parameters of the culture (bacterial biomass concentration and polymer content). As a result, conditions were determined, including controlled fractional dosing of γ-valerolactone for unlimited bacterial growth and polymer synthesis and maximizing the synthesis of copolymers 3HV and 4HV. It has been shown that the highest yields for these monomers in the copolymer are ensured by the use of acrylate to block the reactions of β-oxidation of fatty acids or the replacement of sugars with sodium butyrate. A family of P(3HB-*co*-3HV-*co*-4HV) copolymers with different ratios of monomers: 3HV (from 7.3 to 23.4 mol.%) and 4HV (from 1.9 to 4.7 mol.%) was synthesized at the bacterial biomass concentration (8.2 ± 0.2 g/L), and the PHA content (80 ± 2%) was comparable with the growth on sugars in the control. The physicochemical properties of copolymers and solven casting films obtained from samples of synthesized copolymers with different ratios of monomers have been studied. It has been established that regardless of the ratio of monomers in the copolymer including the content of monomers 3HV and 4HV, all the studied samples of P(3HB-*co*-3HV-*co*-4HV) have reduced values of molecular weight parameters, as well as the degree of crystallinity without a clear relationship with the composition of the monomers. The degree of crystallinity of the samples was below 50% and was in the range of 38–49%. The properties of the films surface of various composition which included the use of AFM for registering roughness, and porosity indices (the number and size of pores, the total area of pores relative to the area of films) were studied. In general, most of the samples showed a decrease in the average pore area and an increase in their number, with a total increase in content of 3HV and 4HV monomers. The roughness indices varied somewhat without a clear relationship with the ratio of monomers in the copolymers. The results obtained make it possible to scale up the productive synthesis of P(3HB-*co*-3HV-*co*-4HV) copolymers using wild type strain, organize the production of samples of this type of degradable PHA for processing into polymer products and conducting multicenter studies.

## Figures and Tables

**Table 1:** Literature data on synthesis and properties of PHA containing 3HV and 4HV when 4HV monomer precursors were added to the culture medium.

Strain, Substrate	X, g/L	PHA, %	3HB	3HV	4HV	M_w_, kDa	Đ	T_melt_, °C	T_degr_, °C	T_g_, °C	C_x_, %	References
*A. eutrophus* H16, 4-hydroxyvaleric acid	-	52	39.9	53.9	6.3	-	-	-	-	-	-	[41]
*A. eutrophus* H16, γ-valerolactone	-	73	41	54.1	4.9	-	-	-	-	-	-	[41]
*A. eutrophus* NCIB 11599, 4-hydroxyvaleric acid	-	45	23.9	67.3	8.8	-	-	-	-	-	-	[42]
*C. necator* USMAA2-4, oleic acid + γ-valerolactone	-	-	8	91	1	510	2.6	95.5	281	−25.1	9.7	[43]
*C. necator* USMAA2-4, oleic acid + γ-valerolactone	-	-	43	55	2	110	3.8	148.9	281	−24.4	7.9	[43]
*C. necator* USMAA2-4, oleic acid + γ-valerolactone	-	-	71	27	2	570	2.9	147.8	275	−13.2	19.7	[43]
*C. necator* USMAA2-4, oleic acid + γ-valerolactone	-	-	90	9	1	280	3.4	145.2	263	−12.8	20.8	[43]
*C. necator* ATCC 17699, glycerol + levulinic acid (3-L fermenter, fed-batch)	58	80	79	18.5	2.5	810	2.2	79 161 175	-	−1.3	42	[44]
*C. necator* ATCC 17699, levulinic acid	-	-	38	55.3	6.7	1060	2.2	60	-	−8.3	34	[44]
*R. eutropha* H16, glucose + levulinic acid (2-L fermenter)	15.5	81.2	46.1	53.9	-	-	-	-	-	-	-	[45]
*R. eutropha* H16, glucose + levulinic acid	-	-	84	16	-	-	-	150.2	298.3		50.3	[45]
*R. eutropha* H16, glucose + levulinic acid	-	-	47	53	-	-	-	101.9	284.4		51.9	[45]
*Cupriavidus sp.* L7L, levulinic acid	3.9	55	45.1	50.1	4.8	4032	4.1	71.8	256.6	−7.87	-	[46]
*Cupriavidus sp.* L7L, levulinic acid	3.5	60	64.2	33.7	2.1	2825	3.6	92.0	250.5	−0.62	-	[46]
*C. necator* H16 CCM 3726, fructose + levulinic acid	7.3	47.8	84.2	15.8	-	668	1.05	167.2	-	-	-	[47]
*Burkholderia sacchari* DSM 17165, xylose + levulinic acid	3.3	45	57	53	-	3910	2.98	148.3 157.7	-	2 −13		[48]
*Burkholderia sacchari* DSM 17165, xylose + levulinic acid	3.1	32	35	65	-	2774	2.78	99.2	-	−4 −14		[48]
*Burkholderia sacchari* DSM 17165, xylose + glucose + levulinic acid	5.3	49	88	12	-	2542	2.48	168.7		3		[48]
*Burkholderia sacchari* DSM 17165, xylose + glucose + levulinic acid	2.7	30	24	76	-	2930	2.91	103.4		3 −14		[48]

**Table 2:** Physicochemical properties of P(3HB-*co*-3HV-*co*-4HV) with different ratio of monomers.

N	Composition of Monomers, mol.%			M_n_, kDa	M_w_, kDa	Ð	C_x_, %	T_melt_, °C	T_degr_, °C	T_g_, °C	T_cryst_ °C
	3HB	3HV	4HV								
1	89.9	7.3	2.8	45 ± 1	212 ± 9	4.7 ± 0.1	49	145.0/ 168.0	284.1	0.5	54.0
2	88.3	9.4	2.3	51 ± 2	248 ± 29	4.9 ± 0.7	46	147.1/ 162.9	280.4	0.3	71.2 55.9
3	83.7	14.4	1.9	87 ± 6	242 ± 10	2.8 ± 0.1	43	151.0 164.5	270.6	1.1	55.7 58.2
4	81.2	16.3	2.5	59 ± 3	201 ± 10	3.4 ± 0.1	46	166.1	281.2	1.2	57.2 51.8
5	76.9	20.8	2.3	46 ± 2	215 ± 11	4.6 ± 0.1	43	142.0 160.0	275.1	−0.7	68.0
6	71.9	23.4	4.7	70 ± 7	203 ± 15	2.9 ± 0.1	38	150.4 164.8	263.4	−10.6 1.47	54.2 61.5
7	100	0	0	248	620 ± 30	2.5 ± 0.1	74	179.3	279.4	5.3	81.6

**Table 3:** Characteristics of solvent cast films produced from P(3HB-*co*-3HV-*co*-4HV) of different compositions compared to P(3HB-*co*-3HV) and P(3HB).

N	Copolymer Composition, Mol.%			Porosity			Surface Roughness		
	3HB	3HV	4HV	Average Pore Area, µm^2^	Number of Pores, Pores/1000 µm^2^	Total Pores Area, µm^2^/1000 µm^2^	Arithmetic Mean Surface Roughness, (S_a_) nm	Root Mean Square Roughness, (S_q_) nm	Peak-To-Valley Height, (S_z_) nm
P(3HB-*co*-3HV-*co*-4HV)									
1	89.9	7.3	2.8	13.7	24.0	328	202	263	2010
2	88.3	9.4	2.3	248.4	2.6	643	544	699	3994
3	83.7	14.4	1.9	81.7	12.8	1047	388	491	2878
4	81.2	16.3	2.5	17.9	29.6	530	206	266	1768
5	76.9	20.8	2.3	2.6	133.7	353	208	260	1738
6	71.9	23.4	4.7	5.5	88.0	488	317	397	2407
P(3HB-*co*-3HV)									
1	85.0	15.0	0	1.0	260	260	373	471	3260
2	35.0	65.0	0	1.5	430	645	489	445	4295
P(3HB)									
1	100.0	0	0	0.02	38.0	0.76	154	180	1256

## Data Availability

All data is available in the paper.

## Supplementary Materials

The following supporting information can be downloaded at: https://www.mdpi.com/article/10.3390/polym14194226/s1, Figure S1: Strategies of adding γ-valerolactone (and sodium acrylate) in split portions during cultivation of *Cupriavidus necator* B-10646 on fructose or sodium butyrate.
