# Supplementary material for: Biosynthesis and Properties of a P(3HB-co-3HV-co-4HV) Produced by Cupriavidus necator B-10646
Source: Polymers (Basel). 2022 Oct 9;14(19):4226. doi: 10.3390/polym14194226 (PMC9570873; doi:10.3390/polym14194226)
Supplement: Supplementary file 1 [file polymers-14-04226-s001.zip › polymers-1926614-supplementary.pdf]

# Biosynthesis and Properties of a P(3HB-*co*-3HV-*co*-4HV) Produced by *Cupriavidus necator* B-10646

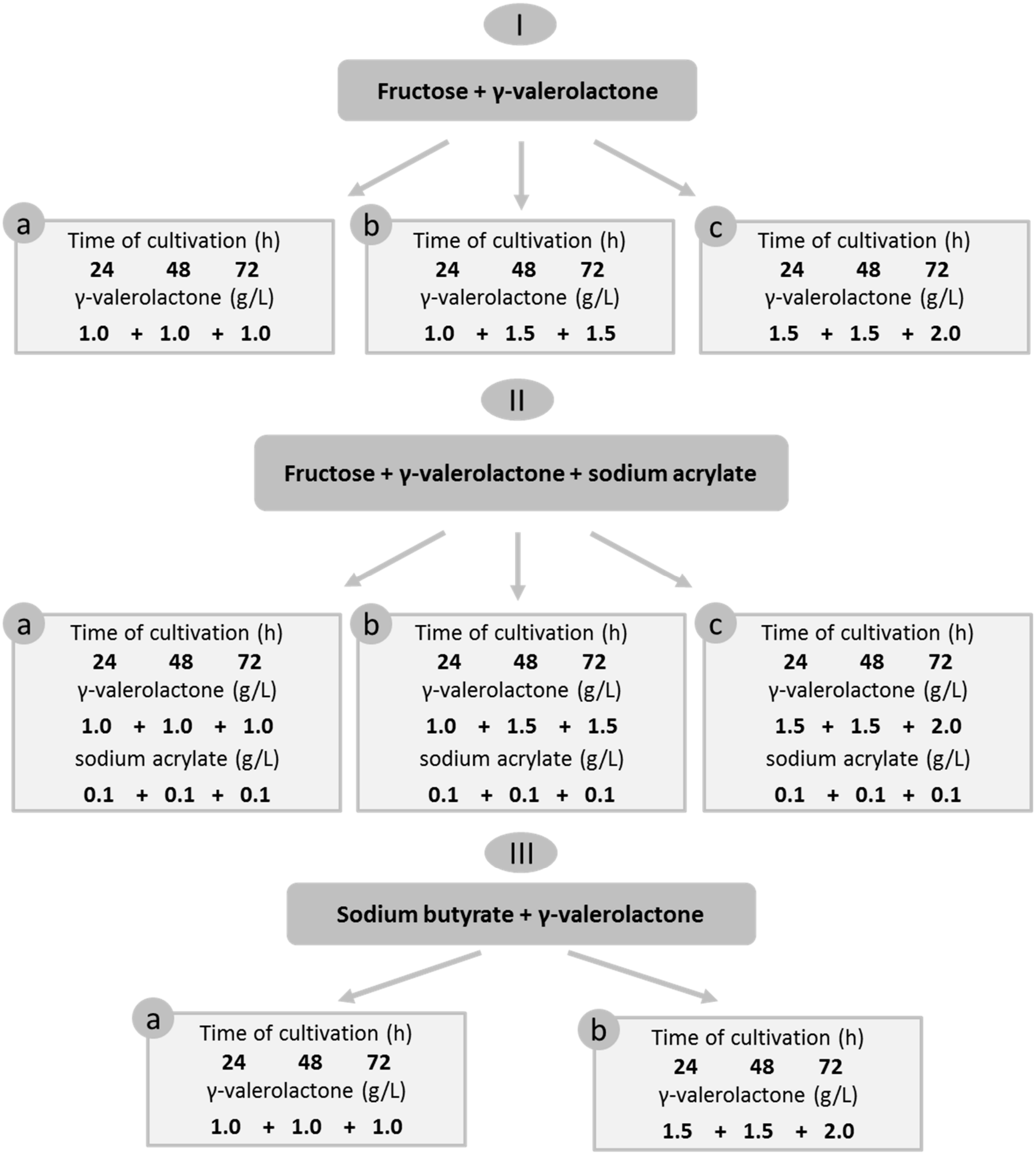


**Figure S1.** Strategies of adding γ-valerolactone (and sodium acrylate) in split portions during cultivation of *Cupriavidus necator* B-10646 on fructose or sodium butyrate.
