# Supplementary figures and images for: Biosynthesis and Properties of a P(3HB-co-3HV-co-4HV) Produced by Cupriavidus necator B-10646
Source: Polymers (Basel). 2022 Oct 9;14(19):4226. doi: 10.3390/polym14194226 (PMC9570873; doi:10.3390/polym14194226)

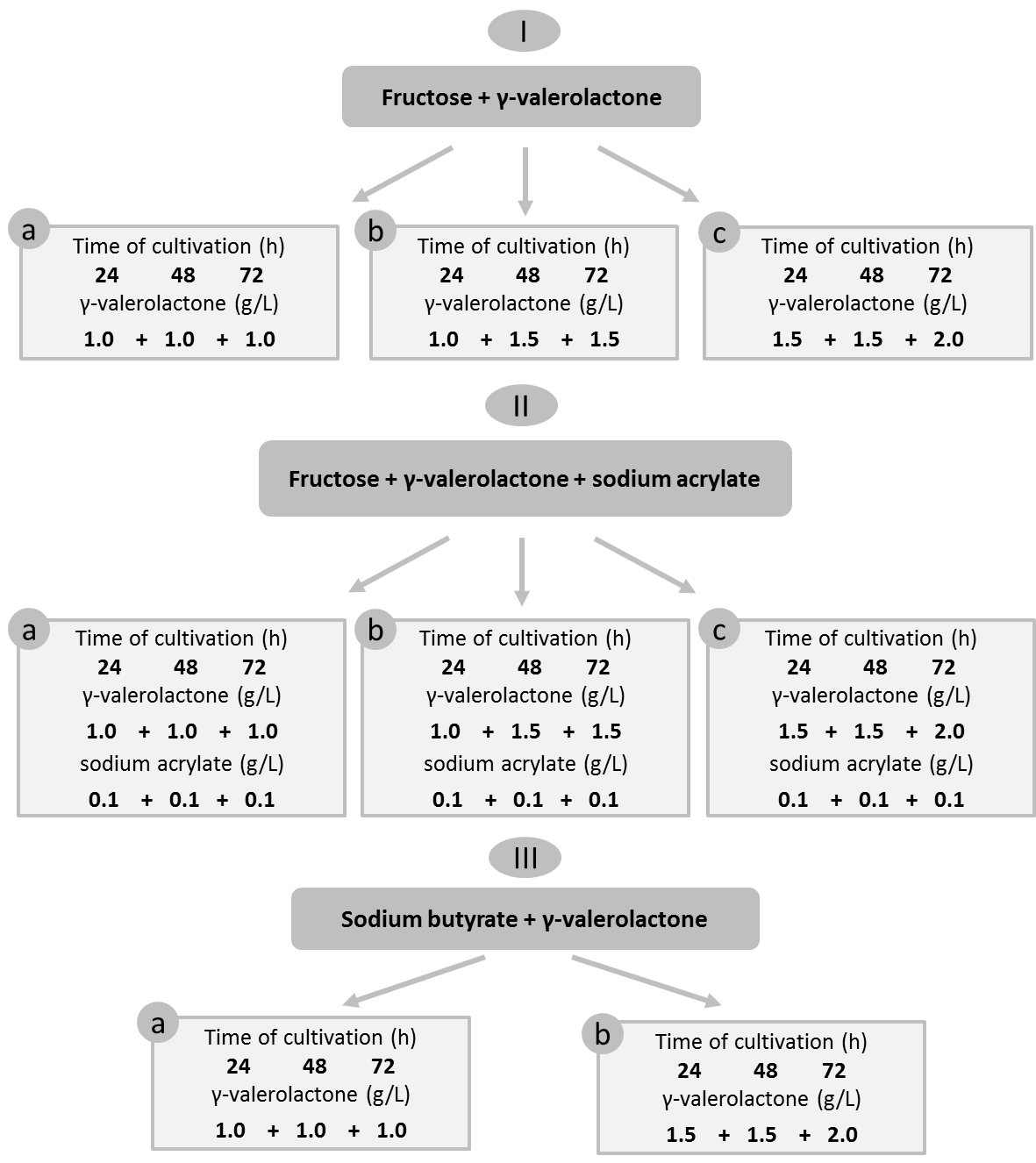

Supplement: Supplementary file 1 [file polymers-14-04226-s001.zip › polymers-1926614-supplementary/polymers-1926614-Figure S1.tif]
